# The Adaptable Binding Cleft of RmuAP1, a Pepsin-like Peptidase from *Rhodotorula mucilaginosa*, Enables the Enzyme to Degrade Immunogenic Peptides Derived from Gluten

**DOI:** 10.3390/biom15121725

**Published:** 2025-12-11

**Authors:** Yu-Han Zhang, Chia-Liang Lin, Menghsiao Meng

**Affiliations:** 1 Doctoral Program in Microbial Genomics, National Chung Hsing University, 250 Kuo-Kuang Road, Taichung 40227, Taiwan; yuhan0307@dragon.nchu.edu.tw; 2 Academia Sinica, 128 Academia Road, Section 2, Nankang, Taipei 11529, Taiwan; 3 Graduate Institute of Biotechnology, National Chung Hsing University, 250 Kuo-Kuang Road, Taichung 40227, Taiwan; 4 Graduate Institute of Biochemistry, National Chung Hsing University, 250 Kuo-Kuang Road, Taichung 40227, Taiwan

**Keywords:** *Rhodotorula mucilaginosa*, celiac disease, enzyme therapy, molecular dynamics, protein–peptide interaction, induced-fit mechanism

## Abstract

Celiac disease (CD) is an autoimmune disorder triggered by pepsin-resistant, gluten-derived immunogenic peptides (GIPs) in genetically predisposed individuals. Enzyme therapy targeting GIPs has been suggested as a complementary practice to a gluten-free diet to help reduce the symptoms of CD. Here, we present the crystal structure of RmuAP1, a pepsin-like aspartic protease from *Rhodotorula mucilaginosa*, which effectively degrades the toxic 33-mer and 26-mer GIPs under postprandial gastric conditions (pH 3.0–6.0). RmuAP1 has a canonical fold characteristic of the aspartic protease subfamily A1; however, it features a distinct flap and a flexible loop structure. Compared to pepsin, RmuAP1 accommodates the tetrapeptides PQQP and PQPQ, motifs frequently repeated on GIPs, via an adaptable binding cleft. Molecular dynamics (MD) simulations have shown that RmuAP1 stably engages these ligands, maintaining both the catalytic water in position and a closed flap conformation, primarily through ligand-induced remodeling of the S1′ pocket. In contrast, pepsin neither binds these ligands effectively nor achieves a catalytically competent conformation. Structural comparisons and dihedral analysis further support an induced-fit mechanism underlying RmuAP1’s pocket remodeling. Together, this study clarifies the structural basis for RmuAP1 to hydrolyze GIPs, emphasizing the potential of RmuAP1 as a platform for developing enhanced oral peptidase for CD patients through protein engineering approaches.

## 1. Introduction

Celiac disease (CD) is an autoimmune disorder triggered by gluten consumption in genetically susceptible individuals who carry the human leukocyte antigen (HLA) alleles DQ2 or DQ8 [1,2,3]. Its hallmark pathological features include villous atrophy and crypt hyperplasia in the small intestine, which lead to nutrient malabsorption and various gastrointestinal symptoms [4,5]. Due to the generation of autoantibodies, such as anti-tissue transglutaminase (TG2) IgA, CD can also manifest in multiple organs and systems, resulting in neurological issues, orthopedic complications, and dermatitis herpetiformis [6,7,8].

Currently, no effective pharmacological treatment exists for CD. The only available management is strict, lifelong adherence to a gluten-free diet (GFD) [9]. While this may seem simple, it can be challenging in practice, as consuming even 20 mg of gluten daily can trigger persistent intestinal inflammation [10]. Hidden gluten is prevalent in processed foods, including canned soups, hot dogs, salad dressings, seasonings, sauces, and more. Additionally, cross-contamination with gluten in food processing facilities further complicates adherence to a gluten-free diet. These factors make maintaining a strict GFD particularly difficult for CD patients.

Gluten is a group of water-insoluble proteins found in wheat, barley, and rye flour. The alcohol-soluble fraction of gluten is called prolamin because of the rich proline and glutamine content. Depending on the grain source, prolamin is named as gliadins (wheat), hordeins (barley), and secalins (rye) [5]. Due to the high proline content, prolamin is resistant to complete digestion in the stomach by pepsin [11,12]. Some resulting partially digested peptides, called gluten-derived immunogenic peptides (GIPs), exhibit immunogenic properties. Among these, the 33-mer peptide from α2-gliadin and the 26-mer peptide from γ5-gliadin are well-documented for their toxicity [11,13].

Upon reaching the lamina propria of the intestine, GIPs undergo modification by TG2, which converts specific glutamine residues into glutamate [14]. These modified GIPs are then efficiently presented to anti-gluten CD4+ T cells by dendritic cells displaying HLA-DQ2 or HLA-DQ8 proteins. Once activated, these T helper cells release pro-inflammatory cytokines, including IFN-γ and IL-21, which drive tissue inflammation [15]. Chronic inflammation ultimately leads to villous atrophy and crypt hyperplasia. Additionally, activated T helper cells stimulate anti-gluten and anti-TG2 B cells, which produce anti-deamidated GIP and anti-TG2 antibodies, respectively. Given this pathological mechanism, administering an effective peptidase that can hydrolyze GIPs before they reach the duodenum could prevent the following GIP-induced immune activation [16]. Theoretically, such peptidases could function as an oral supplement taken after meals to help alleviate CD symptoms. Patients with another subset of gluten-triggered disorder, known as non-celiac gluten sensitivity, might also benefit from taking such peptidases.

Driven by patient demand and commercial potential, various gluten-degrading proteases from different biological sources have been identified and characterized [17]. Notable examples are described below. Latiglutenase is a 1:1 formulation of recombinant peptidases EP-B2 and SC-PEPs: EP-B2, a cysteine protease from barley, and SC-PEP, a serine protease of the S9 family derived from *Sphingomonas capsulata*. A Phase II clinical trial (ClinicalTrials.gov No: NCT03585478) showed that high doses of Latiglutenase could reduce gluten-induced intestinal damage and alleviate symptoms in CD patients [18]. TAK-062 is a computationally designed protease derived from a serine protease of the S53 family originally identified in *Alicyclobacillus sendaiensis*. A Phase I clinical trial (ClinicalTrials.gov: NCT03701555) confirmed its safety and tolerability for treating CD, but similarly required high doses [19]. Neprosin is a glutamate-class prolyl endopeptidase originally discovered in the pitch fluid of the carnivorous plant *Nepenthes × ventrata*. The recombinant enzyme, produced in human Expi293F cells, efficiently degrades the immunogenic 33-mer peptide both in vitro and in the mouse stomach [20]. Despite these promising developments, challenges remain—notably, the need to lower the required therapeutic doses and to improve the efficiency and scalability of peptidase production.

We recently identified a pepsin-like peptidase from *Rhodotorula mucilaginosa*, designated RmuAP1. When heterologously expressed in *Yarrowia lipolytica* Po1g, RmuAP1 was accurately processed, and the mature catalytic domain was efficiently secreted into the culture medium [21]. The mature enzyme exhibits strong hydrolytic activity against the immunogenic 33-mer and 26-mer peptides within a pH range of 6.0 to 3.0—mirroring the physiological pH shift in the stomach after food intake—highlighting its potential as a promising candidate for oral enzyme therapy in CD.

Despite its sequence similarity to RmuAP1, pepsin cannot hydrolyze the 33-mer and 26-mer peptides [21]. Understanding the structural basis for this difference is particularly interesting. In this study, we determined the crystal structure of mature RmuAP1 in complex with pepstatin A. Additionally, we conducted molecular dynamics (MD) simulations to examine the interaction dynamics between RmuAP1 and pepsin with two tetrapeptide ligands, PQPQ and PQQP, which are recurring motifs within GIPs. Our analyses reveal that RmuAP1 undergoes ligand-induced remodeling of the S1′ pocket, enabling stable and catalytically favorable ligand binding. In contrast, pepsin exhibits limited pocket adaptability, leading to suboptimal binding or ligand dissociation. These findings uncover the mechanistic basis for RmuAP1’s capability in the hydrolysis of GIPs and provide a foundation for structure-guided engineering, aimed at enhancing its therapeutic potential for CD.

## 2. Materials and Methods

### 2.1. Enzyme Preparation

The mature RmuAP1 tagged with a hexahistidine at the C terminus was produced by *Y. lipolytica* Po1g and further purified from the culture medium by immobilized metal affinity chromatography as described previously [21]. The purified peptidase was then concentrated to 5 mg/mL, and simultaneously, the buffer was replaced with 20 mM sodium acetate, pH 5.0, using the Amicon Ultracel 10K filters (Millipore, Burlington, MA, USA).

### 2.2. Crystallization

Peptidase crystallization was conducted using the hanging-drop vapor diffusion method with the Crystal Screen2™ kit (Hampton Research, Aliso Viejo, CA, USA). In each well of a 24-well sitting-drop plate, a 1 µL drop of the mature RmuAP1 (5 mg/mL) was mixed with 1 µL of a specific screening solution, and the mixture was allowed to equilibrate against 200 µL of the same screening solution in the reservoir at 20 °C. Following screening, crystals that formed in the screening solution containing 0.1 M NaH_2_PO_4_, 0.1 M KH_2_PO_4_, 0.1 M 2-(N-morpholino)ethanesulfonic acid (pH 6.5), and 2 M NaCl were selected for further X-ray diffraction analysis.

### 2.3. Crystal Structure Determination

Crystals were transferred to a cryoprotectant solution consisting of the screening solution and 25% glycerol, then flash-cooled in liquid nitrogen before data collection. X-ray diffraction data were collected at −173 °C using an EIGER2 X 16M area detector on the TPS 07A beamline at the National Synchrotron Radiation Research Center (Hsinchu, Taiwan) [22]. The synchrotron X-ray wavelength and crystal-to-detector distance were 0.979060 Å and 180 mm, respectively. Data were recorded with an oscillation angle of 0.5° and an exposure time of 0.008 s per frame. The dataset was processed using the HKL-2000 program package, and the protein structure was determined by molecular replacement (MR) with Phaser, included in the Phenix. The model was manually adjusted in Coot and further refined using Phenix.

### 2.4. Protein-Ligand Docking Simulation

Two protein receptor structures were used: RmuAP1, determined from the crystal structure resolved in this study, and pepsin, retrieved from the Protein Data Bank (PDB ID: 1PSO). Tetrapeptide substrates (PQPQ or PQQP) were generated using the “Small Molecules” module in Biovia Discovery Studio (DS) 2016. The binding region was defined based on the RmuAP1–pepstatin A complex, with the “SBD Site Sphere” parameters set to (−3.966397, 5.646897, 24.806000) and a radius of 9 Å, forming a spherical docking region. CDOCKER was used to dock the substrate peptides to the receptor’s catalytic site. Docking accuracy was assessed using “CDOCKER ENERGY” values, with ligand poses showing root mean square deviation (RMSD) < 2.0 Å from the co-crystallized pepstatin A considered reliable.

### 2.5. MD Simulation

MD simulations of the protein–tetrapeptide complexes were performed using the GROMACS 2020.6 software package [23] with the CHARMM36 force field [24,25]. To investigate the binding mode and structural characteristics of the protein (RmuAP1 or pepsin) with the tetrapeptide ligand, the system setup was based on previous studies [26,27,28]. To check for the protonation in titratable residues under pH 3.0 conditions, the pKa was calculated using the H++ server [29]. Each protein–ligand complex was placed in a periodic dodecahedron water box (90 × 90 × 90 Å^3^) and solvated with TIP3P water. Na^+^ and Cl^−^ ions were added randomly to neutralize the system and maintain a physiological ion concentration (0.15 M). Energy minimization was conducted via the steepest descent method (<1000.0 kJ/mol/nm). Equilibration under NVT and NPT ensembles ran for 100 ps each, with a 12 Å cutoff for electrostatic and van der Waals interactions. Simulations for a 100 ns span were performed in triplicate with a 2-fs integration time step (50 million steps). Post-simulation analyses included RMSD, radial distribution functions g(r), minimum distance (Mindist), and hydrogen bonding. Structural analysis and visualization were performed using VMD 1.9.1, PyMOL 3.1.0, and ChimeraX 1.8.

### 2.6. Binding Free Energy Calculations

Molecular mechanics/Poisson–Boltzmann surface area (MM/PBSA) calculations were performed using the set of scripts provided with gmx_MMPBSA 1.6.3. A single-trajectory protocol was employed: only the protein–ligand complexes were subjected to MD simulations, and no separate simulations of the free receptor or free ligand were carried out. To fortify the structural metrics and assess convergence, the binding free energy ($\Delta G_{bind}$) estimates were calculated from snapshots extracted at regular intervals from two segments of each 100 ns trajectory, namely the first 10 ns (0–10 ns) and the last 10 ns (90–100 ns). For each snapshot, the effective free energies of the complex, receptor, and ligand were evaluated within the gmx_MMPBSA framework, and $\Delta G_{bind}$ was obtained as:

(1) $$\Delta G_{bind}=G_{Complex}-G_{receptor}-G_{ligand}$$

## 3. Results and Discussion

### 3.1. Crystal Structure of RmuAP1

The mature RmuAP1 was successfully crystallized in the presence of the aspartic protease inhibitor pepstatin A. The crystal structure of the RmuAP1 complexed with pepstatin A was determined at a resolution of 1.78 Å by X-ray crystallography. The crystals belonged to the orthorhombic space group P22_1_2_1_. The atomic coordinate file was deposited in the PDB with code 9UF9. Data collection and refinement statistics are summarized in Table S1.

**Figure 1:**
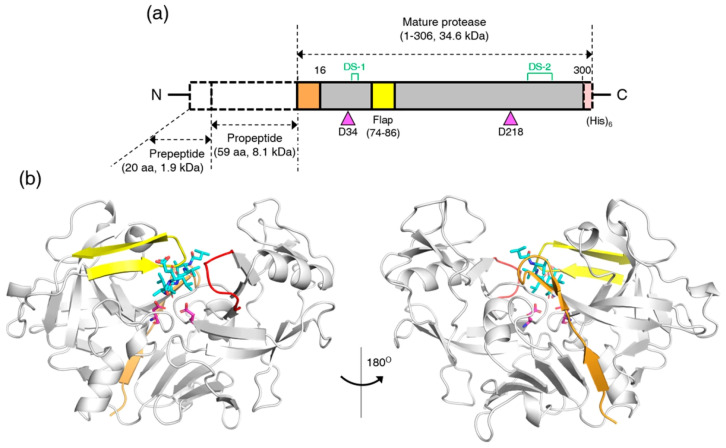
Full-length sequence and crystal structure of RmuAP1. (**a**) Schematic representation of full-length RmuAP1, including the prepeptide, propeptide, and catalytic domain. (**b**) Ribbon representation of the RmuAP1–pepstatin A complex in two views rotated 180° around the *y*-axis. The catalytic dyad residues (Asp34 and Asp218) are highlighted as magenta sticks, while pepstatin A is shown as cyan sticks. Key structural regions are color-coded: the flap region (yellow), the flexible loop (red), and the N-terminal segment occupying the exosite (Ser1–Leu15, orange).

The full-length amino acid sequence of RmuAP1 consists of three major regions (Figure 1a): (1) a prepeptide composed of 22 amino acids, (2) a propeptide comprising 59 amino acid residues, and (3) a catalytic domain consisting of 300 residues. To facilitate subsequent purification, a hexahistidine tag (His_6_) was introduced at the C-terminus of the protein. The structure of mature RmuAP1 comprises two topologically similar β-barrel domains connected by a six-stranded antiparallel β-sheet (Figure 1b), forming a canonical fold characteristic of the aspartic protease A1 subfamily [30,31]. Pepstatin A binds within the central catalytic cleft, where the dyad residues (Asp34 and Asp218) are positioned. A β-hairpin flap (Leu74-Gly86) sits atop the active site, acting as a dynamic lid that likely modulates pepstatin A binding. Additionally, a flexible loop (Asp297-Ala302) contributes to ligand accommodation by adapting its conformation. The first two β-strands and the adjacent loop (Ser1-Leu15) occupy the exosite, reminiscent of the N-terminal segment in the protease IrCD1, which participates in pH-dependent exosite binding and conformational inhibition [32]. Whether mature RmuAP1 employs a similar regulatory mechanism to IrCD1 remains to be elucidated.

**Figure 2:**
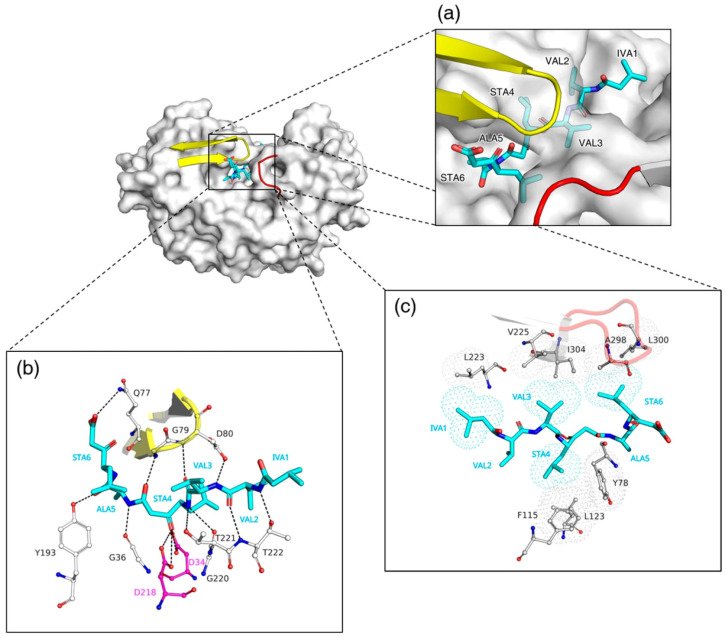
Crystal structure of RmuAP1–pepstatin A complex. (**a**) Top view of pepstatin A positioned within the ligand-binding cleft. (**b**) Hydrogen-bonding interactions between RmuAP1 and pepstatin A. Involved residues are shown as white sticks, and hydrogen bonds are depicted as black dashed lines. (**c**) Hydrophobic interactions between RmuAP1 and pepstatin A, with the interacting residues displayed using a surface dot representation (PyMOL dot mode).

Pepstatin A, a naturally occurring peptide inhibitor, has the structure Iva-Val-Val-Sta-Ala-Sta (where Iva denotes isovaleryl and Sta denotes statine). The central statine residue contributes a non-cleavable pseudopeptide that mimics the tetrahedral transition state of peptide bond hydrolysis, rendering pepstatin A a potent inhibitor of pepsin-like peptidases. In the complex of RmuAP1 with pepstatin A, the inhibitor adopts an extended conformation across the catalytic cleft, occupying subsites from S4 to S2′ (Figure 2a). The binding of pepstatin A to RmuAP1 is accompanied presumably by a positional shift of the β-hairpin flap toward the cleft. At the tip of the flap, residues Gln77, Gly79, and Asp80 form hydrogen bonds with pepstatin A (Figure 2b). Additional hydrogen-bond interactions involve residues Tyr193, Gly36, Asp218, Asp34, Thr221, and Thr222. Hydrophobic residues, including Leu223, Val225, Ala298, Leu300, and Ile304, further stabilize the binding of pepstatin A through hydrophobic interactions (Figure 2c).

### 3.2. Structural Comparison of RmuAP1 and Pepsin

**Figure 3:**
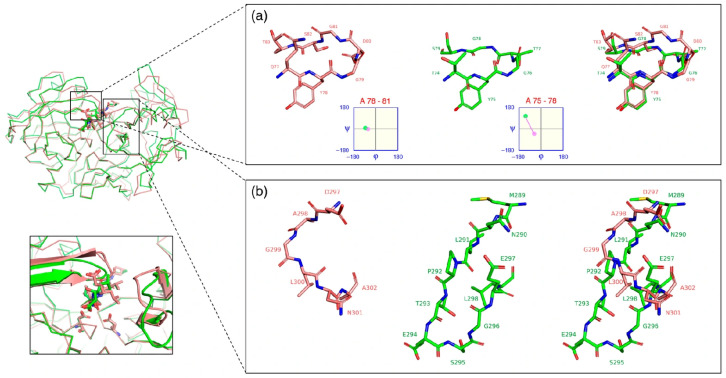
Structural comparison of RmuAP1 and pepsin. Superimposed crystal structures of RmuAP1 (salmon) and pepsin (green) reveal key conformational differences. (**a**) Close-up view of the flap regions, highlighting the distinct β-turn topologies. Ramachandran plots for the corresponding β-turn residues (Ala78-Gly81 in RmuAP1 and Ala75-Gly78 in pepsin) are shown below each structural panel, illustrating differences in backbone dihedral angles. (**b**) Close-up view of the flexible loop regions located along one of the flanks of the catalytic cleft.

To explore the structural basis for functional divergence, the crystal structures of RmuAP1 and human pepsin (PDB ID: 1PSO) were superimposed (Figure 3). Despite the conserved overall fold of the aspartic protease A1 family, two notable differences were observed: the conformation of the flap region (Figure 3a) and the length of a flexible loop (Figure 3b). The flap of RmuAP1 adopts a type I β-turn (Tyr78-Gly81), defined by characteristic dihedral angles: φ/ψ = −64.6°/−30.9° at residue i + 1 and −86.5°/8.1° at residue i + 2. Specific hydrogen bonding interactions were identified between the NH group of Tyr78 and the carbonyl oxygen of Ser82, as well as between the hydroxyl group of Tyr78 and the indole nitrogen (NE1) of Trp41. In contrast, the region of pepsin adopts a type VIII β-turn spanning residues Tyr75-Gly78, with dihedral angles of φ/ψ = −56.7°/−40.1° at residue i + 1 and −127.1°/107.9° at residue i + 2. Notably, a one-residue shortening in the pepsin flap (Gly78 and Ser79 in pepsin, corresponding to Gly81 and Thr83 in RmuAP1) further alters the local conformation (Figure 3a). Previous studies have demonstrated that insertions or deletions within the flap region can significantly perturb the hydrogen-bonding network and alter substrate specificity, particularly affecting interactions at the S1 subsite. For instance, insertion of a serine residue between Gly78 and Ser79 in the pepsin flap disrupts the native interaction network and alters its substrate preference [33].

Another notable structural difference is the flexible loop along one of the flanks of the catalytic cleft (Figure 3b). In RmuAP1, this flexible loop (Asp297-Ala302) comprises only six residues, whereas the corresponding region in pepsin (Leu291-Ser299) contains ten residues. The length of the flexible loop may directly influence its conformational flexibility and local spatial accommodation, thereby affecting substrate binding and catalytic dynamics. Previous studies have shown that a longer flexible loop confers increased conformational freedom and facilitates binding to a broader range of substrates, but at the cost of reduced catalytic precision [34]. In contrast, a shorter loop may enhance substrate specificity and catalytic efficiency.

### 3.3. Structural Basis for Distinct Binding Modes of Pepstatin A in RmuAP1 and Pepsin

**Figure 4:**
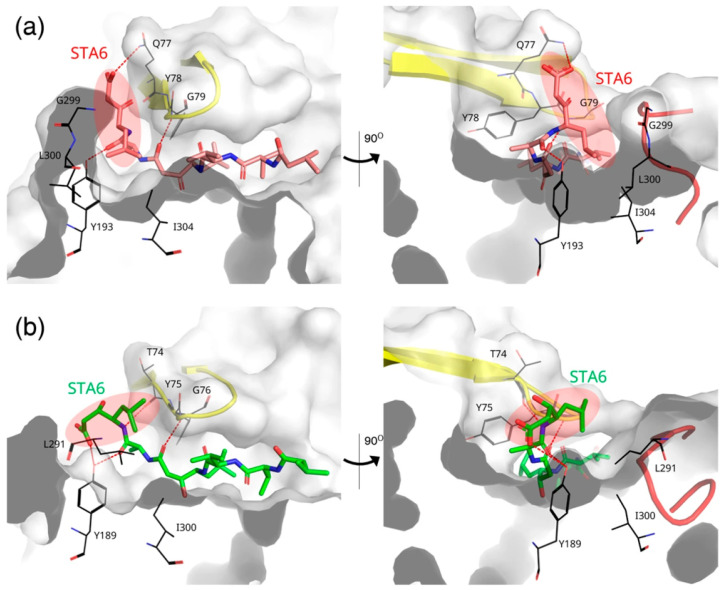
Comparative analysis of the pepstatin A conformation in the binding pockets of RmuAP1 and pepsin. (**a**) RmuAP1–pepstatin A complex; (**b**) pepsin–pepstatin A complex. Each complex is depicted in two orientations rotated 90° around the *y*-axis. Pepstatin A is shown as sticks (salmon in RmuAP1, green in pepsin), with the Sta6 moiety highlighted by a red ellipse. The flap regions are rendered as yellow ribbons. Residues involved in hydrogen bonding with pepstatin A are displayed as black sticks, and the specified hydrogen bonds are represented by red dashed lines.

To further investigate the functional implications of the observed structural differences, we compared the complexed structures of RmuAP1–pepstatin A and pepsin–pepstatin A. In the RmuAP1–pepstatin A complex, the omega torsion angle (ω) between Ala5 and Sta6 of pepstatin A is rotated by 59.7° relative to that in the pepsin complex, repositioning the Sta6 residue toward the flap region (Figure 2b and Figure 4a). This reorientation enables the formation of a unique hydrogen bond between the carboxylate oxygen of Sta6 and the NE2 atom of Gln77 in RmuAP1. In addition, Leu300 in RmuAP1 engages in an alkyl–alkyl interaction with the leucyl side chain of Sta6, contributing to the formation of a long and narrow pocket site, allowing vertical insertion of Sta6 into the active-site cleft (Figure 2c and Figure 4a). In contrast, in the pepsin–pepstatin A complex, the carboxylate oxygen of Sta6 is oriented downward, forming a hydrogen bond with the phenolic hydroxyl group of Tyr198 (Figure 4b).

Crystal structures of these complexes offer critical insights into the molecular interactions involved in ligand binding. These differences in interaction networks reflect the structural variations in the flap and flexible loop regions between RmuAP1 and pepsin. These structural insights establish a foundation for subsequent MD simulations, which will provide a more detailed understanding of how RmuAP1 accommodates ligand binding in comparison to pepsin.

### 3.4. Catalytic Geometry of RmuAP1–Ligand Complexes

**Figure 5:**
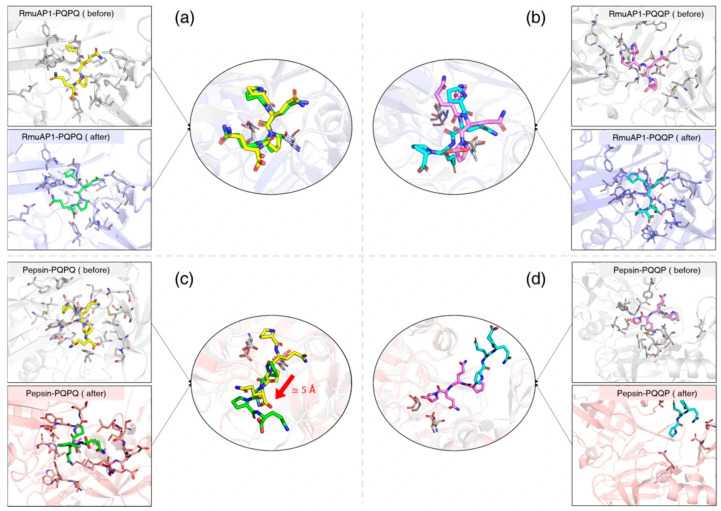
Superimposed diagrams of the peptidase-ligand complex structure before and after MD simulation. Figures show the superposition results of RmuAP1–PQPQ (**a**), RmuAP1–PQQP (**b**), pepsin–PQPQ (**c**), and pepsin–PQQP (**d**) complexes, respectively. Before simulation, the ligands were represented by yellow (PQPQ) and violet (PQQP) stick models, while after simulation, the ligands were represented by green (PQPQ) and cyan (PQQP) stick models.

**Figure 6:**
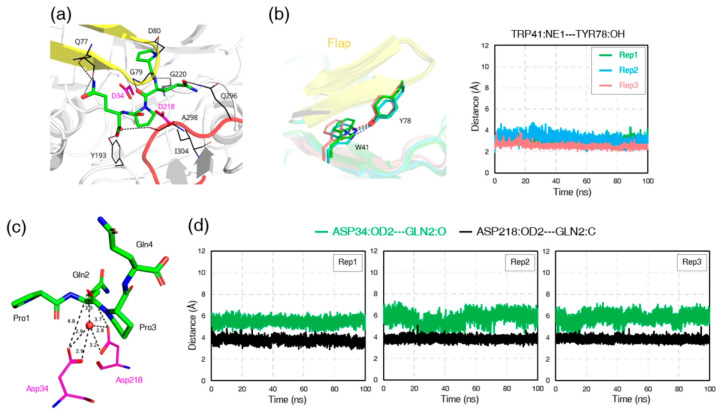
Structural dynamics and interactions of the RmuAP1–PQPQ complex during MD simulations. (**a**) Schematic representation of the representative structure of the RmuAP1–PQPQ complex after convergence. (**b**) Distance fluctuations between Trp41 (NE1) and Tyr78 (OH) across three independent MD replicates. (**c**) Interactions between the catalytic dyad, the PQPQ ligand, and a bridging water molecule, shown in the representative structure. (**d**) Time-dependent distances between the carboxylic oxygen atom OD2 of the catalytic dyad residues and the carbonyl carbon (C) and oxygen (O) atoms of the P1 residue (Gln2 in PQPQ), measured across three independent MD simulation replicates.

**Figure 7:**
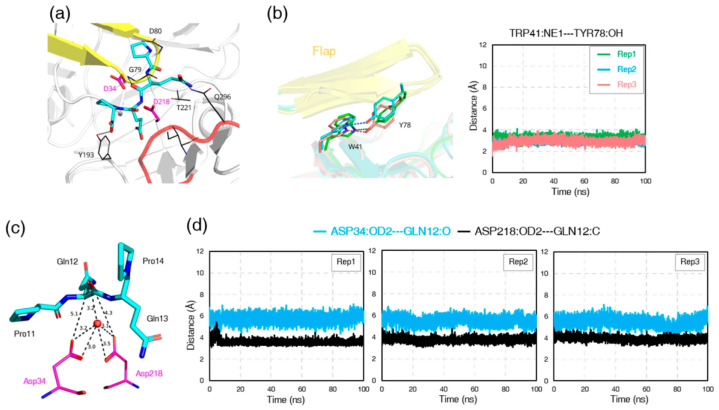
Structural dynamics and interactions of the RmuAP1–PQQP complex during MD simulations. (**a**) Schematic representation of the representative structure of the RmuAP1–PQQP complex after convergence. (**b**) Distance fluctuations between Trp41 (NE1) and Tyr78 (OH) across three independent MD replicates. (**c**) Interactions between the catalytic dyad, the PQQP ligand, and a bridging water molecule, shown in the representative structure. (**d**) Time-dependent distances between the carboxylic oxygen atom OD2 of the catalytic dyad residues and the carbonyl carbon (C) and oxygen (O) atoms of the P1 residue (Gln12 in PQQP), measured across three independent MD simulation replicates.

Previously, we demonstrated that RmuAP1, but not pepsin, catalyzes the hydrolysis of the 26-mer and 33-mer GIPs [21]. To explore the structural basis for this differential proteolytic activity at the molecular level, the tetrapeptides PQPQ and PQQP—repeating motifs found in the 26-mer and 33-mer GIPs—were selected as ligands for constructing peptidase–ligand complex models. Considering that an ideal oral peptidase for CD patients should function optimally in the gastric environment (pH < 4.0) and that RmuAP1 exhibits significant catalytic activity at pH 3.0 [21], we conducted 100 ns MD simulations at pH 3.0. This approach enabled us to evaluate the dynamic behavior of intermolecular interactions under conditions that closely mimic the acidic milieu of the stomach. Key parameters such as stereochemical geometry, system energy profile, interatomic distance, and Mindist were monitored throughout the simulations (Figure 5, Figure 6 and Figure 7 and Figures S1–S6). The structure of RmuAP1 used in the simulations was obtained from the present study, whereas the structure of pepsin was retrieved from the Protein Data Bank (PDB ID: 1PSO). The PQPQ and PQQP ligands were modeled as residues 1–4 and 11–14 of chain I, respectively.

To evaluate conformational changes within the peptidase–ligand complexes, structural snapshots at the beginning of the simulation (0 ns) and representative structures derived from the post-simulation were superimposed (Figure 5). In both the RmuAP1–PQPQ and RmuAP1–PQQP simulations, the backbone conformation of the binding pocket remained stable, with minimal overall deviation throughout the trajectories (Figure S1). Specifically, the average Mindist between the RmuAP1 and the ligand during the simulation was 1.8 ± 0.2 Å for PQPQ in the RmuAP1–PQPQ complex, and 1.6 ± 0.1 Å for PQQP in the RmuAP1–PQQP complex.

Subsequent analysis of hydrogen-bond interactions revealed distinct but persistent networks stabilizing both complexes. In the RmuAP1–PQPQ complex, the side chains of PQPQ engaged residues Gln77, Gly79, and Asp80 in the flap region and Gln296 and Ala298 in the flexible loop, forming an extended hydrogen-bonding network around the catalytic cleft (Figure 6a; Table S2). In the RmuAP1–PQQP complex, the side chains of PQQP primarily contacted Gly79 and Asp80 (flap region) and Gln296 (flexible loop), thereby maintaining the peptide in a catalytically favorable orientation (Figure 7a; Table S2). To quantify the stability of these networks, we calculated hydrogen-bond occupancies over three independent 100 ns trajectories. Several hydrogen bonds between the ligand and the flap or loop showed high occupancies (>70%; Table S2), consistent with persistent interactions. Additionally, throughout the RmuAP1–ligand simulations, the flap region remained in a closed conformation, stabilized by a persistent hydrogen bond between the hydroxyl group of Tyr78 and the NE1 atom of Trp41 (Figure 6b and Figure 7b), with average distances of 2.9 ± 0.2 Å (with occupancy 92.57%) in the RmuAP1–PQPQ complex and 3.0 ± 0.1 Å (with occupancy 97.84%) in the RmuAP1–PQQP complex. This interaction likely facilitates proper substrate positioning between the catalytic dyad residues, promoting an optimal geometry for hydrolysis.

Further catalytic geometry analysis revealed a catalytically relevant water molecule consistently present throughout the RmuAP1–tetrapeptide ligand simulations (Figure 6c and Figure 7c). This water molecule bridged the carboxylate oxygen atoms of the catalytic dyad residues (Asp34 and Asp218) and the carbonyl oxygen of P1 residue (Gln2 in PQPQ, Gln12 in PQQP). In the RmuAP1–PQPQ complex, the distances between the carboxylate oxygens of the dyad and the carbon (C) and oxygen (O) atoms of Gln2 were 3.8 ± 0.1 Å and 5.7 ± 0.2 Å, respectively (Figure 6d). Similarly, in the RmuAP1–PQQP complex, the corresponding distances to Gln12 were 3.8 ± 0.1 Å and 5.6 ± 0.2 Å (Figure 7d). This arrangement formed a classic aspartic protease catalytic water network essential for peptide bond hydrolysis [11]. The stable positioning of this water molecule, as confirmed by radial distribution function (RDF) analysis and system energy profiling, supports its proposed role as a nucleophile to initiate the hydrolytic reaction (Figures S2 and S3). Quantitative analysis of the hydrogen-bonding network revealed that a water-mediated bridge connecting Asp34, Asp218, and the P1 residue was present in 84.5% and 71.7% of bound-state frames for the RmuAP1–PQPQ and RmuAP1–PQQP complexes, respectively. In both complexes, the catalytic water molecule formed an average of 2–3 hydrogen bonds with the catalytic dyad and the P1 residue, establishing a coordination geometry consistent with a nucleophilic water poised for attack. By contrast, in pepsin, the tetrapeptide rapidly dissociated from the active site, allowing solvent molecules to occupy the pocket; the bridging configuration occurred in <1% of the trajectory, preventing a meaningful time-averaged occupancy estimate. To fortify the structural metrics, time-windowed the binding free energy ($\Delta G_{bind}$) estimates were calculated for the first 10 ns (0–10 ns) and the last 10 ns (90–100 ns) of each 100 ns trajectory (Table 1).

### 3.5. MD Simulation of Pepsin–Ligand Complexes

Comparative simulation analyses were performed on pepsin in this study. Molecular docking followed by MD simulations was conducted for both pepsin–PQPQ and pepsin–PQQP complexes. In contrast to the RmuAP1 complex, both pepsin–ligand systems exhibited greater structural variability within the binding region, as indicated by larger Mindist fluctuations (Figures S4 and S5). Notably, both ligands dissociated from the binding cleft before the end of the 100 ns simulations.

In the pepsin–PQPQ complex, PQPQ was partially retained in the catalytic cleft during the first 10 ns. However, by 10 ns, the ligand had drifted approximately 5 Å away from the catalytic dyad (Asp32 and Asp215) (Figure S6a). We found that the average Mindist between the pepsin and the PQPQ ligand during the simulation was 5.7 ± 3.7 Å. Although the water molecule required for nucleophilic attack remained coordinated with the dyad (Figure S6b,c), it failed to adopt a catalytically favorable geometry to initiate the hydrolytic catalysis.

Furthermore, the distances between the catalytic dyad residues and the carbon and oxygen atoms of the Gln2 residue in PQPQ increased substantially, reaching 24.9 ± 11.4 Å and 24.8 ± 12.3 Å, respectively (Figure S6d), further supporting the conclusion that the dyad was unable to facilitate productive catalysis.

In the pepsin–PQQP simulations, the ligand began drifting from the active site as early as 5 ns and was fully dissociated by 20 ns in replicates 1 and 2. In replicate 3, complete dissociation occurred slightly later, at around 40 ns (Figure S5c). The average Mindist of PQQP was 6.5 ± 3.5 Å, and the maximum distance between the catalytic dyad and the ligand reached 56 Å, indicating poor accommodation within the catalytic cleft (Figure S5d).

Flap dynamics also contributed to ligand instability. The hydrogen bond between Tyr75 and Trp39 (corresponding to Tyr78 and Trp41 in RmuAP1) was unstable, and the flap adopted an open conformation during ligand dissociation. This impaired flap closure likely hindered effective ligand retention, consistent with previous observations on the dynamic behavior of pepsin-like aspartic proteases [35]. The binding free energy ($\Delta G_{bind}$) analysis was not performed for the pepsin–tetrapeptide systems due to the absence of any long-lived or well-defined binding poses.

### 3.6. Ligand Binding to RmuAP1 or Pepsin After Simulation

**Figure 8:**
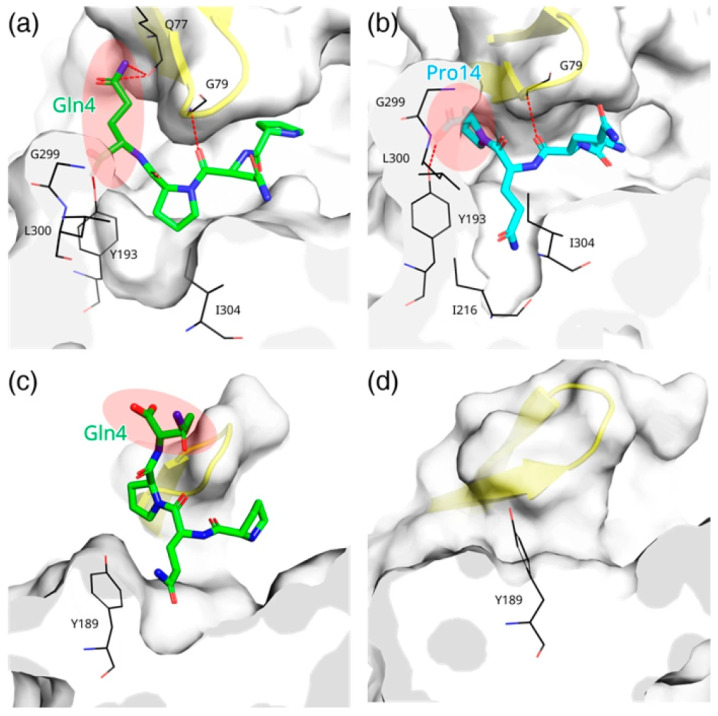
Comparison of peptidase–ligand interactions at the end of the MD simulation. Snapshots of the RmuAP1–PQPQ (**a**), RmuAP1–PQQP (**b**), pepsin–PQPQ (**c**), and pepsin–PQQP (**d**) complexes after MD simulations. The flap region is highlighted in yellow ribbons. The P2′ position (Gln4 in PQPQ; Pro14 in PQQP) is marked with a red ellipse. Substrates are shown as sticks, with PQPQ in green and PQQP in cyan. Key residues interacting with the ligands are displayed as black sticks, and hydrogen bonds are represented by red dashed lines.

The tetrapeptide ligands complexed with RmuAP1 after simulation were structurally aligned with pepstatin A in the RmuAP1–pepstatin A complex. In the RmuAP1–PQPQ complex, the ligand PQPQ adopted a binding conformation similar to that of pepstatin A (Figure 8a). The side chain of Gln4 (P2′) is aligned similarly to the Sta6 moiety of pepstatin A, positioning itself within the S2′ pocket. The NE2 and OE1 atoms of Gln4 formed hydrogen bonds with the backbone oxygen of Gln77, reinforcing this interaction. Conversely, when PQQP bound, the flexible loop residues Gly299 and Leu300 shifted upward to reshape the pocket edge and form a deep, bowl-like S2′ cavity, accommodating the P2′ residue (Pro14) (Figure 8b). The binding modes of the tetrapeptide ligands to RmuAP1 in triplicate are shown in Figure S7.

Although the pepsin–PQPQ complex underwent pocket remodeling throughout the early simulation period (~10 ns) (Figure 8c), the resulting ligand conformation and positioning within the catalytic site remained suboptimal for catalysis (Figure S6b–d). The ligand shifted during the simulation course, hindering the establishment of a critical binding pose necessary for catalysis. This limitation is even more pronounced in the pepsin–PQQP complex (Figure 8d), where the ligand dissociated from the active site early in the simulation. The inability of pepsin to properly accommodate these ligands may explain why it cannot catalyze the hydrolysis of the 26-mer and 33-mer GIPs.

### 3.7. Ligand-Induced Remodeling of the S1′ Pocket in RmuAP1

During 100 ns MD simulations, we found that binding of tetrapeptide ligands triggered a conformational change in the RmuAP1 binding pocket. According to the induced-fit hypothesis [36], substrate binding can be optimized for substrate containment through conformational changes in catalytic enzymes. To observe this conformational change in the protease, we focused on the remodeling of the S1′ pocket of RmuAP1, which exhibited the most significant conformational change in MD simulations.

**Figure 9:**
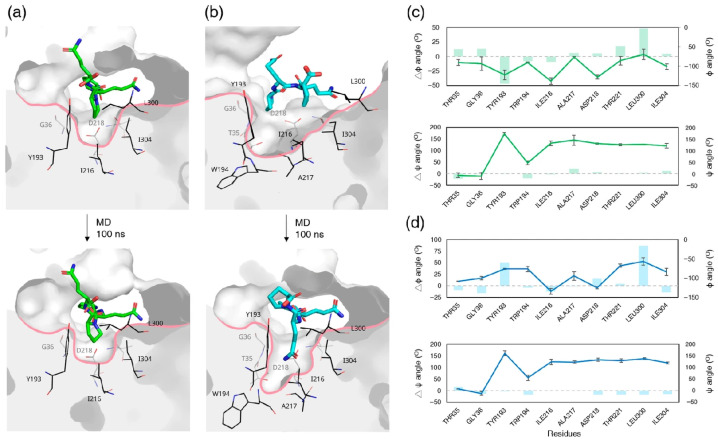
Structural remodeling of the S1′ binding pocket in RmuAP1–ligand complexes before and after MD simulations. Cross-sectional views of the S1′ pocket in the RmuAP1–PQPQ (**a**) and RmuAP1–PQQP (**b**) complexes, shown before and after MD simulations. Pocket-lining residues are shown as black sticks. Substrates are depicted as sticks, colored green for PQPQ and cyan for PQQP. Backbone dihedral angle (φ and ψ) variations in the residues forming the S1′ pocket during the simulation for RmuAP1–PQPQ and RmuAP1–PQQP complexes are illustrated in panels (**c**) and (**d**), respectively. The line in the charts denotes the average angles (φ and ψ) of the indicated residues after MD simulation. The data are mean ± SD in triplicate. The shade bar represents the range of angular changes (Δφ and Δψ) during the simulation.

The simulations revealed distinct remodeling behaviors of the S1′ binding pocket in RmuAP1 in response to different GIP substrates. PQPQ binding required only minimal conformational adjustment (Figure 9a), as the side chain of Pro3 (the P1′ residue) readily fitted into a preformed shallow pocket primarily defined by Ile216. Most pocket residues remained structurally stable (Δφ/ψ < 15°), with only Tyr193 (Δφ = 47.2° ± 12.2°, *p* = 0.00xx) and Leu300 (Δφ = 48.0° ± 13.4°, *p* = 0.0074) undergoing moderate backbone torsional shifts (Figure 9c).

In contrast, binding of PQQP induced a more substantial remodeling of the S1′ pocket to accommodate the longer side chain of Gln13 (Figure 9b). Leu300 exhibited a pronounced backbone torsion (Δφ = 86.4° ± 9.7°, *p* = 0.0015), while Tyr193 also underwent a comparable angular change (Δφ = 50.8° ± 1.5°, *p* = 0.0001), contributing to the deepening of the pocket (Figure 9d). Despite these internal adjustments, the overall pocket aperture remained relatively unchanged, suggesting that RmuAP1 accommodates the ligands not by opening the cleft, but via inward compression mediated by flexible peripheral residues. This highlights the functional importance of targeted side-chain flexibility in fine-tuning substrate compatibility while preserving an optimal catalytic geometry. A bird’s-eye view of the S1′ pocket further reveals that Pro3 in PQPQ occupies a shallow pre-existing cavity. In contrast, binding of Gln13 in PQQP is accompanied by deepening of the pocket through inward displacement of Thr221 and Leu300 (Figure S8).

### 3.8. Ligand-Induced Remodeling of the S1′ Pocket in Pepsin

**Figure 10:**
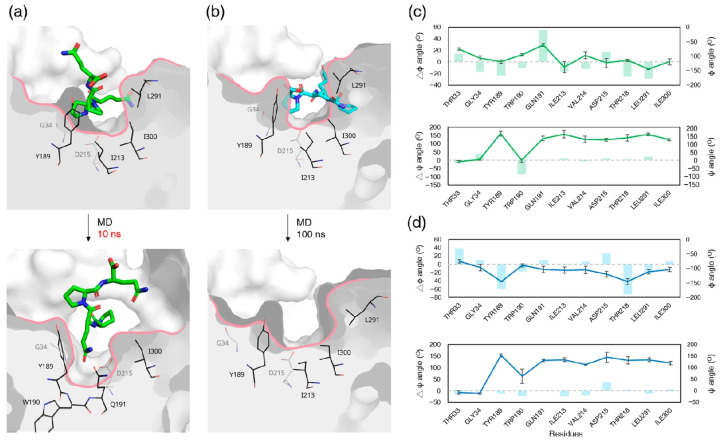
Structural remodeling of the S1′ binding pocket in pepsin–ligand complexes before and after MD simulations. Cross-sectional views of the S1′ pocket in the pepsin–PQPQ (**a**) and pepsin–PQQP (**b**) complexes, shown before and after MD simulations. Pocket-lining residues are shown as black sticks. Substrates are depicted as sticks, colored green for PQPQ and cyan for PQQP. Backbone dihedral angle (φ and ψ) variations in the residues forming the S1′ pocket during the simulation for pepsin–PQPQ and pepsin–PQQP complexes are illustrated in panels (**c**) and (**d**), respectively. The line in the charts denotes the average angles (φ and ψ) of the indicated residues after MD simulation. The data are mean ± SD in triplicate. The shade bar represents the range of angular changes (Δφ and Δψ) during the simulation.

As to the MD simulation of the pepsin–PQPQ complex, the ligand was positioned within the catalytic cleft during the early stages of simulation (~10 ns), transiently adopting a favorable geometry for catalysis (Figure 10a). Several active-site residues exhibited significant backbone dihedral angle changes during the simulation. Tyr189 (Δφ = 24.4 ± 6.2°, *p* = 0.36), Gln191 (Δφ = 54.9° ± 4.7°, *p* value not applicable), Thr218 (Δφ = 25.2° ± 4.4°, *p* = 0.014), and Leu291 (Δφ = 28.7° ± 1.2°, *p* = 0.0074) underwent moderate φ angle shifts, while Trp190 showed a substantial ψ angle shift (Δψ = 86.7 ± 10.3°, *p* = 0.0030), indicating local structural rearrangements around the binding cleft during early ligand accommodation (Figure 10c).

The binding of PQQP failed to induce productive remodeling of the active site of pepsin (Figure 10b). Although several peripheral residues exhibited notable angular shifts, these changes did not lead to a conformation with a catalytically favorable geometry. Specifically, Thr33 (Δφ = 38.7° ± 6.2°, *p* = 0.0082), Tyr189 (Δφ = 59.9° ± 0.1°, *p* = 0.0001), and Thr218 (Δφ = 72.6° ± 10.0°, *p* = 0.0025) displayed outward rotational shifts away from the pocket interior during the simulation (Figure 10d), resulting in a flattened geometry of the S1′ pocket. The disruption of the bowl-like contour of the pocket thereby compromises stable substrate accommodation. Consistently, top-down views of the S1′ pocket show that the bowl-like contour is maintained in the pepsin–PQPQ complex only in the first 10 ns; moreover, it becomes markedly flattened in the pepsin–PQQP complex (Figure S8).

### 3.9. Apo RmuAP1 Simulations Support an Induced-Fit Model for S1′ Pocket Remodeling in RmuAP1

To further support an induced-fit mechanism, we additionally performed three independent 100 ns MD simulations of apo RmuAP1, generated from the RmuAP1–pepstatin A complex structure (PDB 9UF9) by removing the bound inhibitor. At the beginning of these apo simulations, the S1′ pocket adopted a shallow, bowl-shaped conformation (Figure S9a,b), similar to that observed in Figure S8a (the PQPQ-bound state at 0 ns). However, in the absence of a bound ligand, this shallow pocket gradually relaxed into a more flattened geometry over the course of the simulations (Figure S9a,c). This flattening was accompanied by a disengagement of the flexible loop from the flap region (Figure S10a): as the flexible loop opened, Leu300 on the loop moved away from the rim of the pocket and dragged Ile304 outward as well (Figure S10b). In addition, Thr221 shifted away from the pocket rim, causing the two DTG(S/T) motifs of the aspartic protease (Asp34–Thr35–Gly36–Ser37 and Asp218–Thr219–Gly220–Thr221) to move further apart (Figure S10b). The key residues forming the S1′ pocket as a whole also shifted toward the pocket opening (Figure S10c). Thr35 and Trp194 moved upward from the bottom of the pocket toward its rim, collectively diminishing the bowl-like contour. The absence of PQQP-like S1′ conformations in the apo ensemble, together with the rare occurrence of PQPQ-like conformations, supports a predominantly induced-fit remodeling of the pocket driven by binding of the Gln13 side chain in PQQP, rather than a pre-existing population of the deep-pocket state.

## 4. Conclusions

In this study, we elucidated the structural and dynamic features underlying the unique gluten-degrading activity of RmuAP1. Crystallographic analysis revealed that RmuAP1 adopts a canonical A1 fold, featuring a catalytically competent active site composed of a type I β-turn flap and a short hydrophobic flexible loop. MD simulations further demonstrated that RmuAP1 can stably accommodate immunogenic tetrapeptides such as PQPQ and PQQP, maintaining catalytically favorable geometry throughout the simulation. In contrast, pepsin failed to accommodate the ligands during the simulation.

The distinct behaviors of RmuAP1 and pepsin observed in the MD simulations suggest that RmuAP1 exhibits greater structural adaptability than pepsin, enabling it to effectively accommodate GIPs and catalyze the hydrolysis of these immunogenic peptides. Understanding the intrinsic structural features responsible for these distinct behaviors remains an intriguing but challenging task, particularly since both enzymes share conserved pocket-lining residues: Thr35, Gly36, Tyr193, Trp194, Ile216, Ala217, Asp218, Leu300, and Ile304 in RmuAP1, which correspond to Thr33, Gly34, Tyr189, Trp190, Ile213, Ala214, Asp215, Leu291, and Ile300 in pepsin. Therefore, it is tempting to propose that residues that do not directly interact with the substrate (i.e., non-pocket residues), particularly those adjacent to the pocket-lining residues, play an important role in facilitating ligand-induced conformational changes necessary for the accommodation and subsequent catalysis of GIPs. This proposition is supported by prior studies indicating that targeted modifications of residues outside the catalytic site can still significantly alter enzyme catalytic efficiency and substrate preference. Accordingly, strategically modifying the networks involving these non-pocket residues may provide an alternative approach to enhancing the catalytic efficiency of RmuAP1 toward GIPs.

## Figures and Tables

**Table 1:** Average statistics of MM/PBSA calculation.

Protein–Ligand Complexes	ΔG_Bind_ (kJ mol^–1^)
RmuAP1–PQPQ (0–10 ns)	−17.9 ± 3.6
RmuAP1–PQPQ (90–100 ns)	−28.4 ± 6.0
RmuAP1–PQQP (0–10 ns)	−17.6 ± 3.3
RmuAP1–PQQP (90–100 ns)	−28.8 ± 5.5

## Data Availability

All data are included in the Supplementary Materials.

## Supplementary Materials

The following supporting information can be downloaded at: https://www.mdpi.com/article/10.3390/biom15121725/s1, Table S1: Data collection and refinement statistics for the RmuAP1–pepstatin A crystal; Table S2: Analysis of the hydrogen bond network between RmuAP1 and tetrapeptides; Figure S1: Conformational dynamics of RmuAP1 complexed with PQPQ and PQQP during MD simulations; Figure S2: RDF analysis and total energy profiles of the RmuAP1–PQPQ complex across three independent MD simulations; Figure S3: RDF analysis and total energy profiles of the RmuAP1–PQQP complex across three independent MD simulations; Figure S4: MD simulation trajectories for the pepsin–PQPQ complex in triplicate; Figure S5: MD simulation trajectories for the pepsin–PQQP complex in triplicate; Figure S6: Structural rearrangements and catalytic geometry analysis of the pepsin–PQPQ complex; Figure S7: Representative snapshots of RmuAP1–ligand interactions at the end of MD simulations; Figure S8: Top-down views of the S1’ pocket in RmuAP1 and pepsin; Figure S9: Flattening of the S1’ pocket in apo RmuAP1 during MD simulations; Figure S10: Concerted motions of the flap, flexible loop, and S1’ pocket residues in apo RmuAP1.

## Abbreviations

The following abbreviations are used in this manuscript:

CD	Celiac disease
GIPs	gluten-derived immunogenic peptides
TG2	tissue transglutaminase 2
GFD	gluten-free diet
MD	molecular dynamics
DS	Discovery Studio
RMSD	root mean square deviation
Mindist	minimum distance
$\Delta G_{bind}$	binding free energy
MM/PBSA	molecular mechanics-Poisson-Boltzmann surface area
