# Supplementary material for: The Adaptable Binding Cleft of RmuAP1, a Pepsin-like Peptidase from Rhodotorula mucilaginosa, Enables the Enzyme to Degrade Immunogenic Peptides Derived from Gluten
Source: Biomolecules. 2025 Dec 11;15(12):1725. doi: 10.3390/biom15121725 (PMC12730222; doi:10.3390/biom15121725)
Supplement: Supplementary file 1 [file biomolecules-15-01725-s001.zip › biomolecules-4000939-supplementary.pdf]

-Supporting Information

The adaptable binding cleft of RmuAP1, a pepsin-like peptidase from *Rhodotorula mucilaginosa*, enables the enzyme to degrade immunogenic peptides derived from gluten.

Yu-Han Zhang [1,2,4], Chia-Liang Lin [3,*] and Menghsiao Meng [4,*]

1. Doctoral Program in Microbial Genomics, National Chung Hsing University, 250 Kuo-Kuang Road, Taichung 40227, Taiwan; yuhan0307@dragon.nchu.edu.tw (Y.-H.Z.)
2. Academia Sinica, 128 Academia Road, Section 2, Nankang, Taipei 11529, Taiwan
3. Graduate Institute of Biochemistry, National Chung Hsing University, 250 Kuo-Kuang Road, Taichung 40227, Taiwan
4. Graduate Institute of Biotechnology, National Chung Hsing University, 250 Kuo-Kuang Road, Taichung 40227, Taiwan

*Correspondence: mhmeng@dragon.nchu.edu.tw (M.M.); lin.cl@nchu.edu.tw (C.-L.L.)

Table S1. Data collection and refinement statistics for the RmuAP1–pepstatin A crystal

**Crystal**	RmuAP1–pepstatin A
**PDB ID**	9UF9
λ for data collection (Å)	0.979060
**Data collection**	
Space group	$P22_12_1$
**Unit cell dimensions**	
a, *b*, *c* (Å)	41.669, 53.076, 158.594
α, *β*, *γ* (°)	90, 90, 90
Resolution range (Å)	30.00-1.78 (1.89-1.78)
R_{merge} (%)	9.6 (38.8)
Overall $I/\sigma(I)$	8.69 (2.77)
Completeness (%)	95.8 (94.4)
Redundancy	2.2 (2.2)
**Refinement**	
Resolution	28.6-1.8
Reflections (work/test)	33919 (1699)
R_{work}	0.1744
R_{free}	0.2048
RMSD length (Å)	0.006

RMSD angles (°)	0.95
Average B-factor (Å^2)	15.82
**No. of non-H atom**	
Protein	2404
Ligand	48
Water	336
**Ramachandran plot (%)**	
Favored region	98.48
Outliers	0.00

Table S2. Analysis of the hydrogen bond network between RmuAP1 and tetrapeptides

**RmuAP1–PQPQ**		
**Interaction**	**Distance***	**Occupancy (%)**
ASP80:OD2---PRO1:N	2.94±0.10	86.85
GLY220:O---PRO1:O	2.91±0.13	82.00
GLY79:N---GLN2:O	3.17±0.09	75.13
GLN296:NE2---GLN2:OE1	3.07±0.09	82.07
TYR193:OH---GLN4:O	2.66±0.28	89.00
GLN77:O---GLN4:OE1	2.49±0.13	93.33
GLN77:OE1---GLN4:NE2	3.71±0.14	50.67
**RmuAP1–PQQP**		
**Interaction**	**Distance***	**Occupancy (%)**
ASP80:OD2---PRO11:N	3.17 ±0.08	73.31
GLY79:N---GLN12:O	2.89 ±0.15	94.85
GLN296:NE2---GLN12:OE1	3.14 ±0.15	81.33
TYR193:OH---GLN14:N	3.14 ±0.10	68.35

*Mean ± SD were calculated according to three independent 100-ns MD simulations. All distances are measured in Å.

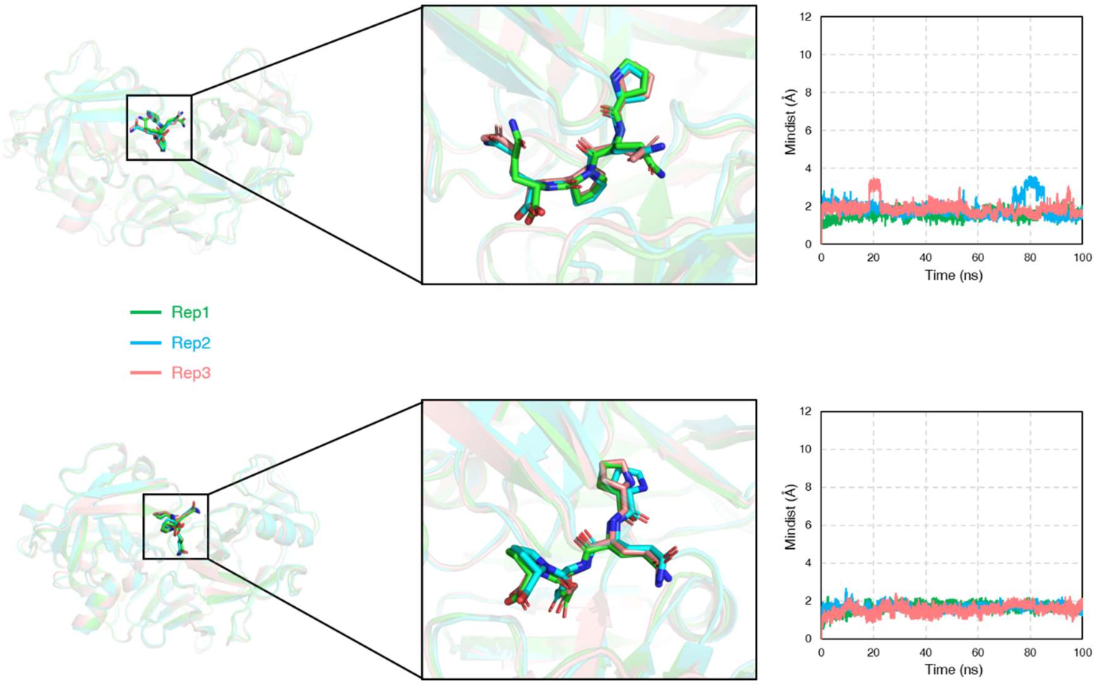

Figure S1. Conformational dynamics of RmuAP1 complexed with PQPQ and PQQP during MD simulations. The top panel shows the RmuAP1–PQPQ complex, and the bottom panel shows the RmuAP1–PQQP complex. RmuAP1 is shown as a cartoon, while the PQPQ and PQQP ligands are depicted as sticks. The right panels show the time-dependent Mindist between RmuAP1 and the ligand throughout the simulation for each replicate. The three replicates are represented in green (Rep1), cyan (Rep2), and salmon (Rep3), respectively.

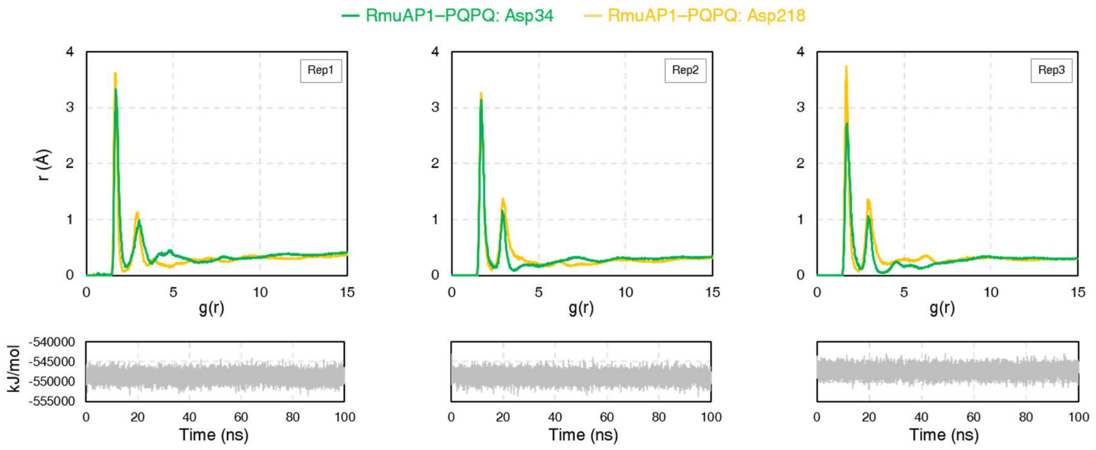

Figure S2. RDF analysis and total energy profiles of the RmuAP1–PQPQ complex across three independent MD simulations. RDF plots show the spatial distribution of a water molecule around the carboxylate oxygens of the catalytic dyad residues Asp34 (green) and Asp218 (yellow). Corresponding total energy profiles are shown below. Total potential energy as a function of simulation time for the RmuAP1–PQPQ complexes (three independent replicas), showing stable fluctuations around a constant mean value after equilibration.

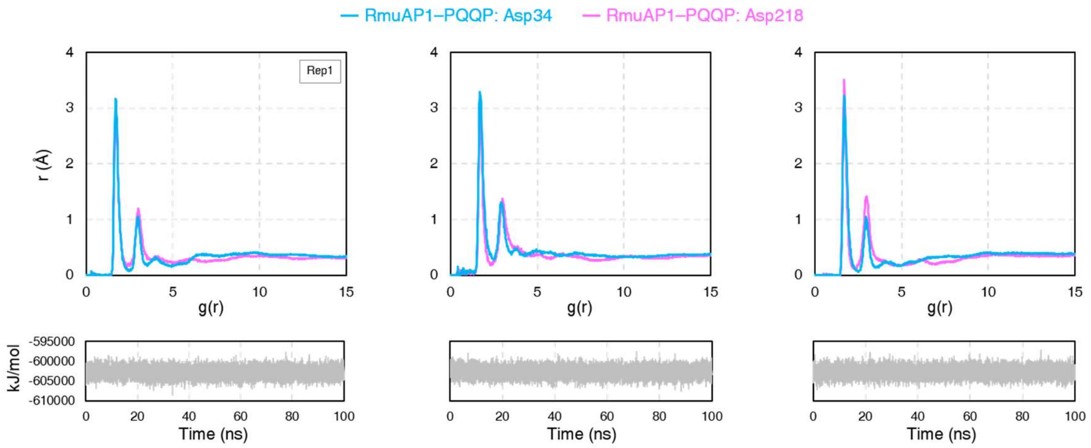

Figure S3. **RDF analysis and total energy profiles of the RmuAP1–PQQP complex across three independent MD simulations.** RDF plots show the spatial distribution of a water molecule around the carboxylate oxygens of the catalytic dyad residues Asp34 (blue) and Asp218 (magenta). Corresponding total energy profiles are shown below. Total potential energy as a function of simulation time for the RmuAP1–PQQP complexes (three independent replicas), showing stable fluctuations around a constant mean value after equilibration.

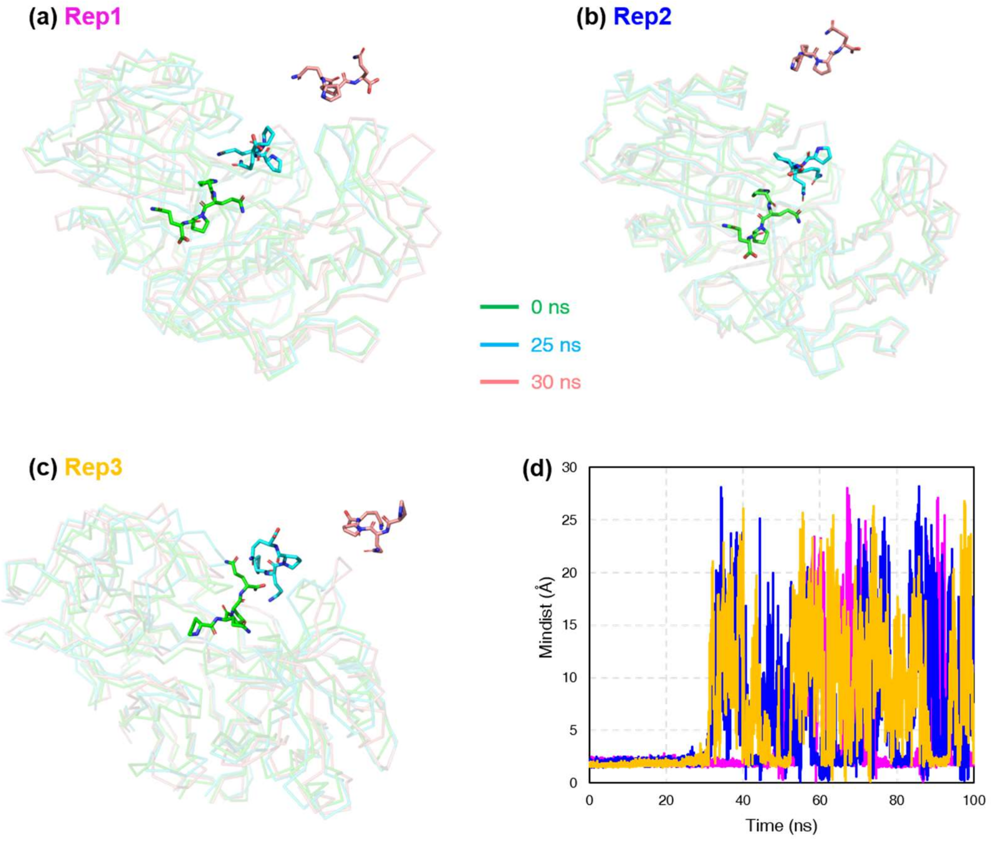

Figure S4. MD simulation trajectories for the pepsin–PQPQ complex in triplicate. (a–c) Structural ensembles of pepsin–PQPQ at selected time points (0 ns, 25 ns, and 30 ns) from three independent MD replicates (Rep1, Rep2, and Rep3, respectively). Protein backbones are shown in ribbon, and the ligand PQPQ is shown in stick. (d) Time-dependent Mindist between pepsin and the PQPQ ligand during each simulation

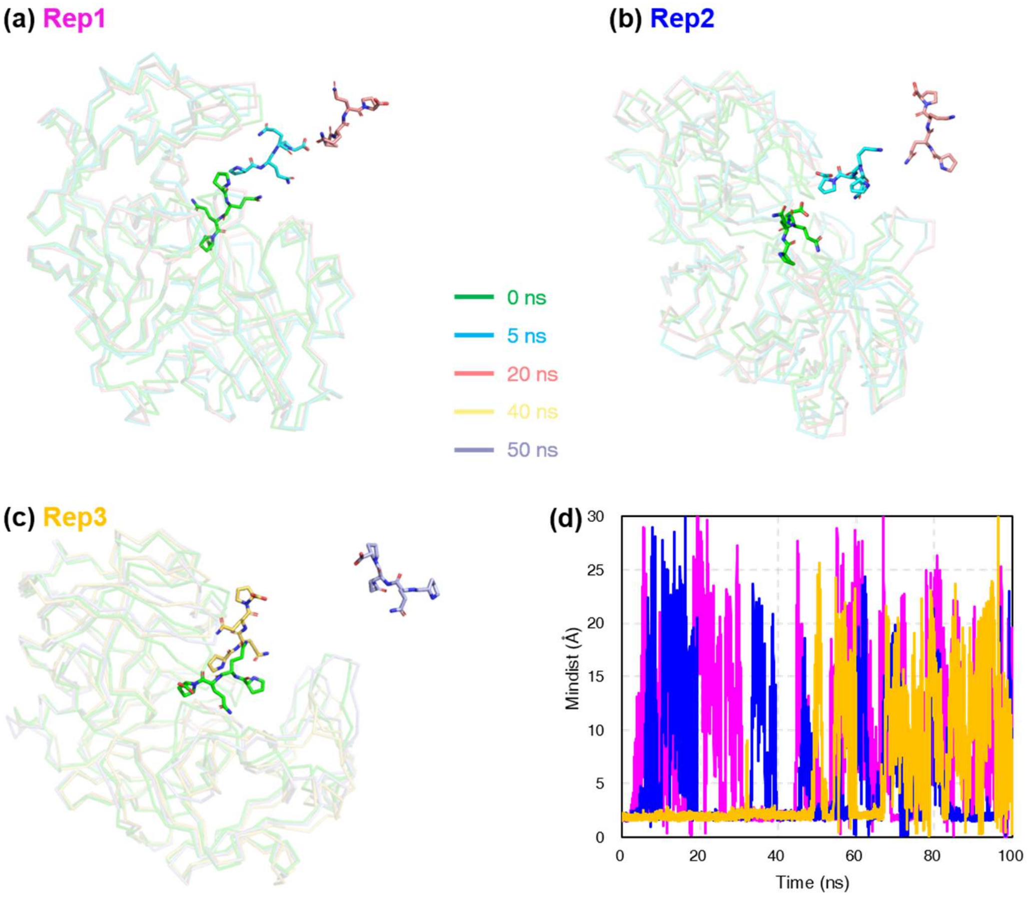

Figure S5. MD simulation trajectories for the pepsin–PQQP complex in triplicate. (a–c) Structural ensembles of pepsin–PQQP at selected time points (0 ns, 5 ns, 20 ns, 40 ns, and 50 ns) from three independent MD replicates (Rep1, Rep2, and Rep3, respectively). Protein backbones are shown in ribbon, and the ligand PQQP is shown in stick. (d) Time-dependent Mindist between pepsin and the PQQP ligand during each simulation

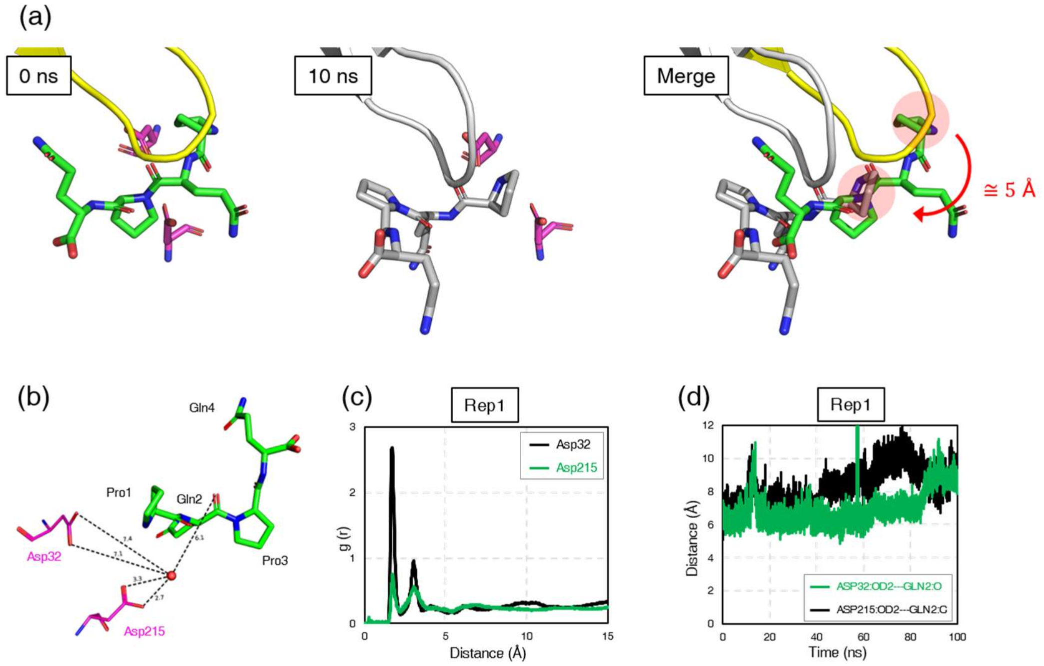

Figure S6. Structural rearrangements and catalytic geometry analysis of the pepsin–PQPQ complex. (a) Conformational changes of the pepsin–PQPQ complex at 0 ns and 10 ns, and their superimposed structure (Merge). (b) Representative structure showing interactions between the catalytic dyad, the PQPQ ligand, and a bridging water molecule at 10 ns. (c) Radial distribution function (RDF) plots showing the spatial distribution of a water molecule around the carboxylate oxygens of the dyad residues Asp32 (black) and Asp215 (green). (d) Time-dependent distances between the carboxylic oxygen atom OD2 of the dyad residues and the carbonyl carbon (C) and oxygen (O) atoms of the P1 residue (Gln2 in PQPQ), based on the pepsin–PQPQ Rep1 trajectory.

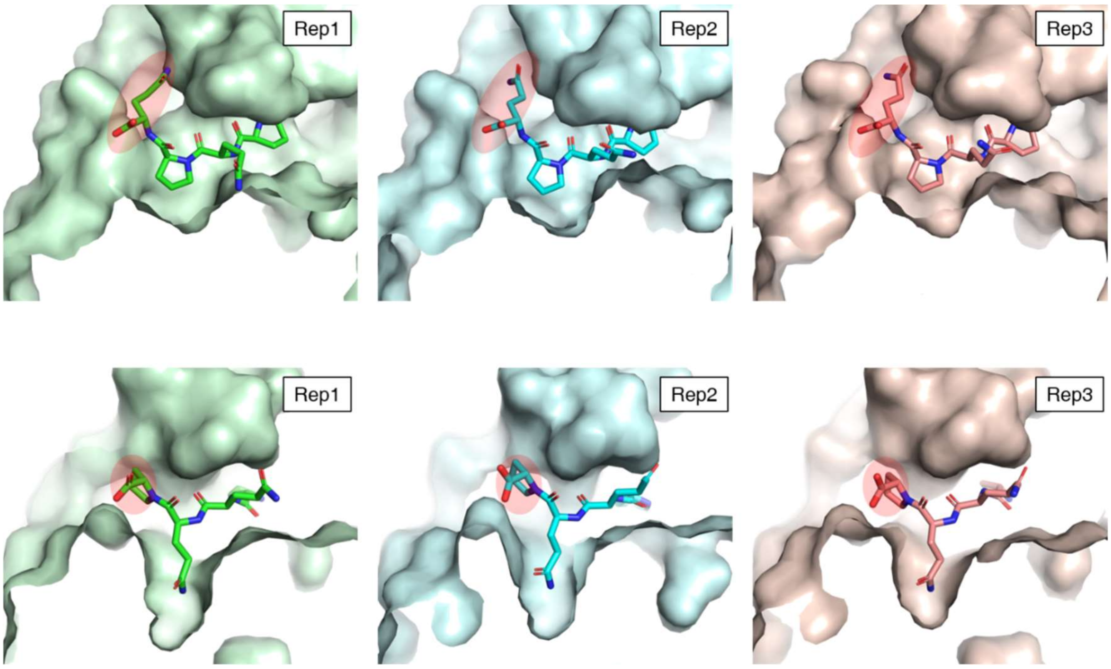

Figure S7. Representative snapshots of RmuAP1–ligand interactions at the end of MD simulations. The top row shows the RmuAP1–PQPQ complex, and the bottom row shows the RmuAP1–PQQP complex. Each panel represents a typical conformation selected from one of the three independent MD simulation replicates (Rep1–Rep3). The P2' position—Gln4 in PQPQ and Pro14 in PQQP—is highlighted with a red ellipse. Protein surfaces are shown in molecular surface representation, and the ligands are shown as sticks.

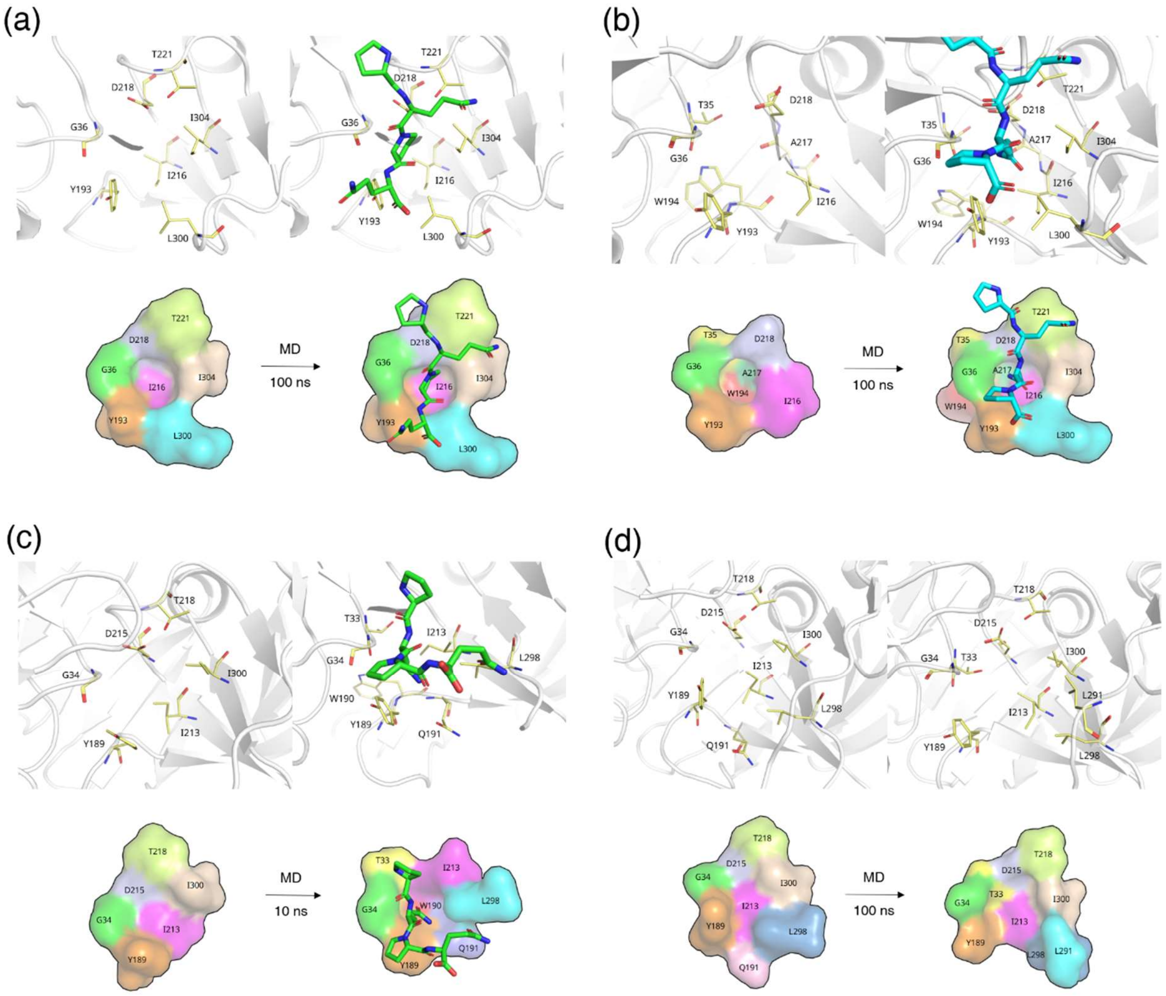

Figure S8. **Top-down views of the S1' pocket in RmuAP1 and pepsin.** Show the S1' pocket of RmuAP1–PQPQ (a), RmuAP1–PQQP (b), pepsin–PQPQ (c), and pepsin–PQQP (d) complexes, respectively. For each complex, the upper panels show the catalytic cleft of the enzyme (gray cartoon) with the bound tetrapeptide (sticks), viewed from the top toward the S1' pocket. The lower panels display the molecular surface of S1' pocket residues, colored and labeled individually to highlight their contributions to the pocket shape.

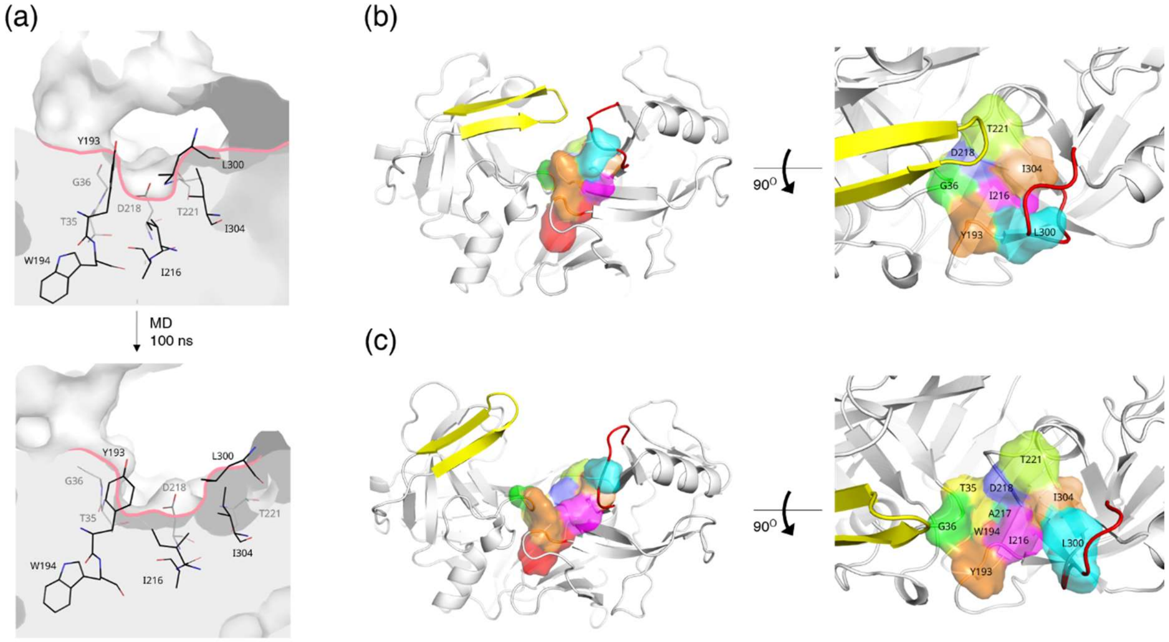

Figure S9. Flattening of the S1' pocket in apo RmuAP1 during MD simulations. (a) Cross-sectional views of the S1' pocket in apo RmuAP1, shown before and after 100-ns MD simulations. Key S1' residues (Thr35, Gly36, Tyr193, Trp194, Ile216, Ala217, Asp218, Thr221, Leu300, and Ile304) are shown as black sticks and labeled. Initial (b) and final (c) states of the overall apo RmuAP1 (gray cartoon) are shown with the flap (yellow), flexible loop (red), and the S1' pocket highlighted as a colored molecular surface, in which individual pocket residues are colored and labeled.

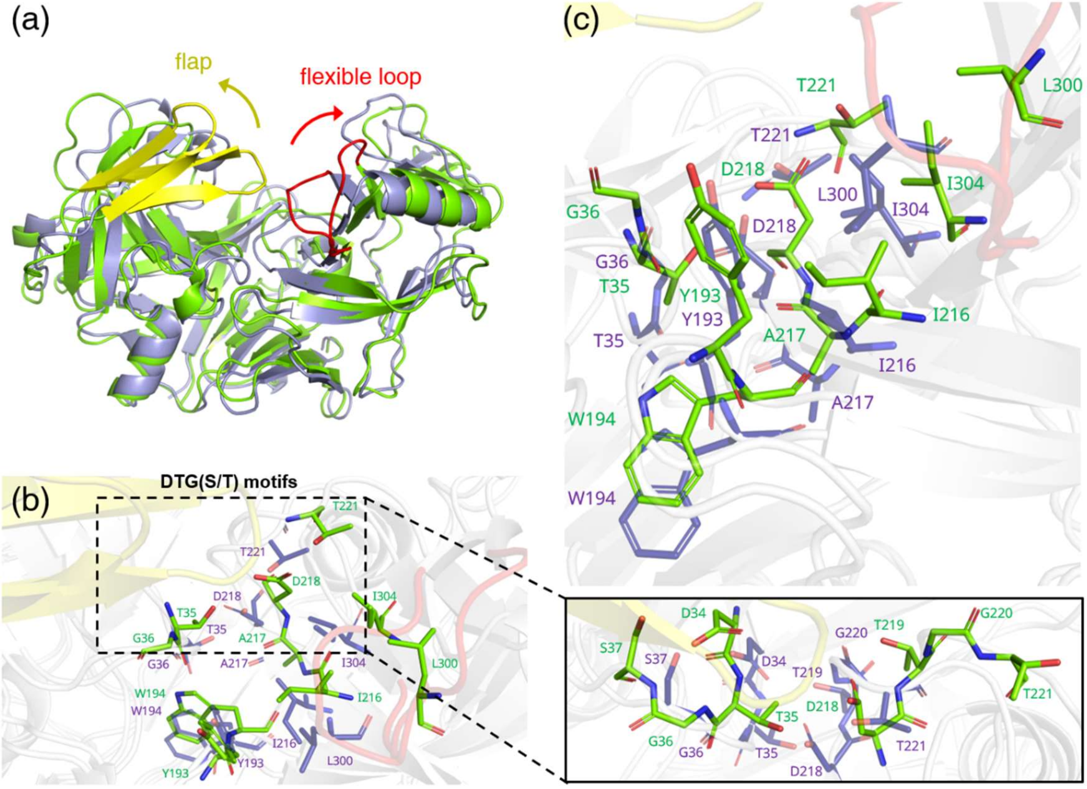

Figure S10. Concerted motions of the flap, flexible loop, and S1' pocket residues in apo RmuAP1. (a) Superposition of representative apo RmuAP1 snapshots before (purple) and after (green) the MD simulations, highlighting the flap (yellow) and the flexible loop (red). Top (b) and side (c) views illustrate close-ups of the S1' pocket, with key residues shown as sticks in the before (purple) and after (green) conformations. In panel (b), a detailed view of the DTG(S/T) motifs (Asp34–Thr35–Gly36–Ser37 and Asp218–Thr219–Gly220–Thr221) is shown.
